# Prevalence of Type II diabetes in District Dir Lower in Pakistan

**DOI:** 10.12669/pjms.323.9795

**Published:** 2016

**Authors:** Sohail Akhtar, Zahid Khan, Muhammad Rafiq, Ajmal Khan

**Affiliations:** 1 Dr. Sohail Akhtar, PhD (UK). Department of Statistics, University of Malakand, Lower dir, KP, Pakistan; 2 Zahid Khan (M.Phil Candidate). Department of Statistics, University of Malakand, Lower dir, KP, Pakistan; 3 Muhammad Rafiq (M.Phil) Department of Statistics, University of Malakand, Lower dir, KP, Pakistan; 4 Ajmal Khan (M.Phil Candidate). Department of Animal Sciences, Quaid-i-Azam University, Islamabad, Pakistan

**Keywords:** BMI, Exercise, Hypertension, Type II diabetes

## Abstract

**Objective:**

To investigate the prevalence of type II diabetes and pre-diabetes and its risk factors in the District Dir Lower Pakistan.

**Methods:**

This study was a population based cross--sectional analysis of 1650 individuals of age 20--80 years, using cluster random sampling technique. After an overnight fast, diabetes and pre-diabetes were analyzed according to the World Health Organization recommendation.

**Results:**

The prevalence of diabetes and prediabetes was 11.1% and 16.0%, respectively. Type II diabetes was found 11.0% in female and 11.2% in male subjects. Stepwise multiple logistic regression showed that growing age, positive family history, body mass index (obesity), hypertension, exercise (less physical activates), education, monthly income, are statistically significant risk factors with type II diabetes.

**Conclusion:**

Our results suggest that type II diabetes has become a main health problem in District Dir Lower and better strategies are required to handle this problem.

## INTRODUCTION

Type II diabetes is a worldwide health problem and affecting more than 415 million individual and expected to reach 642 million individuals by end of 2040.^1^ The prevalence of diabetes has increased over the last few decades along withobesity.^1^-^3^ It has been estimated that the number of individuals with diabetes will increase by 69 percent in developing countries and 20 percent in developed countries between 2010 and 2030.^4^ Another study showed that diabetes is the 4th major reason of death in most developed countries.^5^

In Pakistan, World Health Organization (WHO)reported that 12.9 million individuals suffer from diabetes (10 percent of the population), 9.4 million diabetes patients are diagnosed and 3.5 million people are undiagnosed.^6^ On the other hand, 38 million people (20.5 percent women and 15.9 percent men) have prediabetes. Another study showed that Pakistan stands at number7 among the top ten countries having type II diabetes and projected number4th by the end of 2030.^6^ Furthermore, it is reported that 120,000 people die in Pakistan every year as a result of type II diabetes and its related complications.^7^ All these figures are showing an alarming situation in Pakistan.

A number of research papers have been published to assess the prevalence and to investigate risk factors of type II diabetes in the different cities of Pakistan.^8^-^12^ However, the rapid increase of diabetes rate still needs an improved understanding of risk factors.

Several studies have illustrated that type II diabetes could be delayed or prevented by change in diet and life-style (exercise)in high risk persons.^13^-^16^ Another study showed that comparing standard lifestyle with rigorous lifestyle intervention found a 58% reduction in diabetes incidence.^17^ Thus, the earlier detection of those subjects at high risk for diabetes is essential. Initially, we explore the prevalence of type II diabetes in the District Dir Lower. Secondly, we model the type II diabetes functions of different risk factors to find out the most significant factors for type II diabetes. The risk factors that are included in the analysis are: growing age, body mass index (BMI), hypertension, monthly income, exercise (or physical activities) and smoking, sex, locality (rural/urban).

## METHODS

We used a multi-stage, cluster random sampling technique to select the representative subjects of 20 years age or above in the population. District Dir Lower has a total of 34 union councils. Each union council was considered as single cluster. Initially, six union councils were selected by simple random sampling out of 34 union councils. In the second stage, a total of 1650 subjects (male and female) were selected through simple random sampling procedure from each selected cluster with equal proportion. Voter list was used as sampling frame. Pregnant women were not included in the study. This is because pregnancy may cause the possible impaired glucose tolerance.

Information was collected through a standard questionnaire by trained staff on the demographic characteristics, positive family history of diabetes, educational level, occupation, income level, cigarette smoker (a person who have smoked at least 100 cigarette in life span).^18^ A written consent form was signed by all the individuals before information collection.

Subjects were screened for the Fasting Plasma Glucose (mg per deciliter) at morning, using glucometer(GLUCOCARD®01 ARKRAY USA). They were requested, one day before, to eat or drink anything for at least 8 hours. For diabetes and hypertension, we used the WHO standard.^6^ The subject has type II diabetes if the level of Fasting Plasma Glucose is 126 mg per deciliter or above while prediabetes if their 110 mg per deciliter to 125 mg per deciliter. Note that the test was repeated on the very next day for all those subjects whose level of blood glucose was greater 126 mg/dL to confirm the result. Similarly, if a blood pressure of 140/90 or above was considered hypertension. A subject was declared to be obese if body mass index (BMI) ≥30 kg/m^2^ and over-weight when BMI ≥25 kg/m^2^.

### Ethical approval

All procedures performed in studies involving human participants were in accordance with the ethical standards of the institutional and/or national research committee and with the 1964 Helsinki declaration and its later amendments or comparable ethical standards. In addition, informed consent was obtained from all individual participants included in the study.

### Statistical Analysis

Prevalence of diabetes and prediabetes were measured as percentages. A Chi–square (*χ^2^*) test was considered to assess the trends in the prevalence of diabetes and prediabetes among different age-groups. To study the impact different risk factors on the prevalence of prediabetes and diabetes, we used ordinal logistic regression instead of classical linear regression model. This is because of that the response variable is categorical and has natural order in the categories. The response variable was considered in to three categories: diabetes, prediabetes and normal. On the other hand, independent variables were: age, sex, occupation, monthly income, physical activity or exercise, positive family history of diabetes, hypertension, obesity (BMI). Statistical software SPSS version 20 was used for all the analysis and value with p<0.05 was considered statistical significant.

## RESULTS

**Fig.1:**
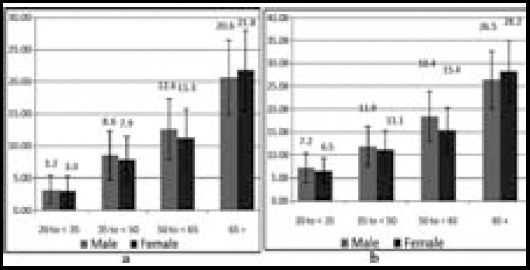
(a) Age-specific prevalences rate of diabetes and (b) Age-specific prevalences rate of prediabetes and I-bar represent the 95% confidence interval.

We investigated the prevalence of diabetes and prediabetes for 1650 subjects within the age range 20—80 years, of which 811 were females and 839 males. The total prevalence of diabetes (previously diagnosed and undiagnosed diabetes) and prediabetes were 11.1% and 15.67%, respectively. The Fig.1 shows the age–specific prevalence of diabetes and prediabetes in the subjects. The age–specific prevalence of diabetes and prediabetes increased significantly with growing age, with P<0.01 (for both groups). But the prevalence of prediabetes and diabetes were not statistical significant according to sex (p=0.06). The prevalence of prediabetes was significantly higher than diabetes in the age– groups: age group 20–34 years (prediabetes 7.38% vs diabetes 3.23%, with p = 0.0078), age group 50—65 years (prediabetes 16.9% vs diabetes 11.9%, with p= 0.043). On the other side there is no significant difference between the prevalence of diabetes and prediabetes in the age-groups: the age group 35–49 years (prediabetes 11.5% vs diabetes 8.25%, with p = 0.138) and age group 65 and above. (prediabetes 27.2% vs 20.7% diabetes, with p= 0.131).

Stepwise multiple ordinal logistic regression results showed that growing age, obesity, positive family history, physical activities (exercise), education, monthly income and hypertension are significantly correlated with a high risk of diabetes. The coefficients of these factors are presented in Table-I. The coefficients. growing age, positive family history, hypertension, obesity (BMI), education, monthly income are negative. This indicate that the higher value of these factors increase the probability (chances) of diabetes. On the other hand, the coefficient value of exercise is positive which indicates that physical activity decrease the risk of diabetes.

**Table-I:** Fitted parameter estimates using stepwise logistic regression.

	*Coefficient*	*Standard Error*	*P value*
Constant 1	-6.173	0.9300	0.000
Constant 2	-8.452	0.9930	0.000
Growing Age	0.104	0.0090	0.000
Family History of Diabetes	1.244	0.3660	0.000
Hypertension	1.843	0.2850	0.000
Education	0.052	0.0200	0.030
Monthly Income	0.0001	0.0001	0.000
Exercise	-0.067	0.0470	0.045
Obesity (BMI)	0.017	0.0330	0.011

## DISCUSSION

The results of the present study are based on the overall prevalence figures of Type II diabetes mellitus from the subject samples. The overall prevalence (prediabetes and diabetes) in this study was found to be 26.1% in both males and females which is higher than earlier studies published in three village of Khyber Pukhtunkhwa^9^ (20.5%), and other provinces such as Sindh^19^ (25%) and Baluchistan^10^ (22%). The high figures of abnormal glucose tolerance (diabetes and prediabetes) found as 25.1% in men and 26.4% in women, and the increase with growing age, is alarming. According to the WHO’s demographic studies, the prevalence of diabetes in Pakistan in 1995, suggested an increase in Type 2 diabetes in Pakistan from 4.3 million in 1995 to 14.5 million in 2025. This will make Pakistan the fourth among top 10 diabetes reporting countries of the world.^20^ This sharp rise in diabetes cases is attributable to many factors, the notable being industrialization and urbanization and a change in lifestyle, both in urban and rural areas.^21^ Our findings also show that the prevalence of prediabetes is greater than diabetes which indicate the potential for further increase in the number of diabetes cases in higher age individuals. Furthermore, our analysis showed that increasing age, hypertension, diabetes history in the family, and monthly earning are the significant risk factor for the prevalence of diabetes. The results are consistent with previously published studies.^8^-^12^ A very known risk factor for Type II diabetes in the Asian population is family aggregation of diabetes, shown by a positive history in first degree relatives. Consanguinity can be related to genetic diabetes as consanguineous marriages is a common practice in this country. Moreover, heavy urbanization and thus subsequent unhealthy lifestyles, malnutrition in mother and thus fetus and genetic factors are also recognized as risk factors for the disease. As a result of the specific life style of the poor class of the country, exercise at the right time is usually reduced among the people.^22^-^23^

Unlike previous published studies,^18^,^24^-^25^ our analysis showed a significant association between educational level and the prevalence of diabetes. This is because that educated peopleare generally less physically active. On the other hand, smoking, gender, occupation were insignificant factors

## CONCLUSION

Our analysis shows that the prevalence of type II diabetes and prediabetes is quite high and urgent preventive measure should be adopted. Diabetes Mellitus needs serious efforts and focused actions for prevention of the disease. Early detection and modification of the risk factors for the development of diabetes complications remains the best available option to deal with this alarming disease. The role of awareness programs and community-based screening campaigns against diabetes should not be overruled. These efforts will surely help reduce the burden of the disease. The results will assist them to understand the effect of associated risk factors of type II diabetes in the area. However, further research is required to find out the association between these risk factors.
